# Immunohistochemistry-derived subtypes of breast cancer distribution in four regions of Ethiopia

**DOI:** 10.3389/fendo.2023.1250189

**Published:** 2023-11-09

**Authors:** Esmael Besufikad Belachew, Adey Feleke Desta, Tewodros Yalew Gebremariam, Dinikisira Bekele Deneke, Senait Ashenafi, Melisachew Mulatu Yeshi, Bizunesh Dires Fenta, Alemwosen T/Hayimanot Alem, Addisu Alemu, Abdo Kedir Abafogi, Tigist Desta, Menberework Chanyalew, Daniel Beshah, Lesley Taylor, Marcus Bauer, Dareskedar Tsehay, Selfu Girma, Daniel Seifu Melka, Tesfaye Sisay Tessema, Eva J. Kantelhardt, Rawleigh Howe

**Affiliations:** ^1^ Biology Department, College of Natural and Computational Sciences, Mizan Tepi University, Mizan, Ethiopia; ^2^ Department of Microbial, Cellular and Molecular Biology, College of Natural and Computational Sciences, Addis Ababa University, Addis Ababa, Ethiopia; ^3^ Non-Communicable Diseases (NCD) Research Directorate, Armauer Hansen Research Institute, Addis Ababa, Ethiopia; ^4^ Department of Pathology, School of Medicine, College of Health Sciences, Tikur Anbessa Specialized Hospital and Addis Ababa University, Addis Ababa, Ethiopia; ^5^ Department of Pathology, School of Medicine, College of Health Sciences, Mekelle University, Mekelle, Ethiopia; ^6^ Pathology Department, Hawassa Referral Hospital, Hawassa, Ethiopia; ^7^ College of Health and Medical Sciences, Haramaya University, Harar, Ethiopia; ^8^ Pathology Department, Jimma University Specialized Hospital, Jimma, Ethiopia; ^9^ Department of Diagnostic Laboratory, Tikur Anbessa Specialized Hospital, College of Health Sciences, Addis Ababa University, Addis Ababa, Ethiopia; ^10^ City of Hope National Medical Center, Duarte, CA, United States; ^11^ Global Health Working Group, Martin Luther University Halle-Wittenberg, Halle (Saale), Germany; ^12^ Institute of Pathology, Martin Luther University Halle-Wittenberg, Halle (Saale), Germany; ^13^ Department of Biochemistry, Division of Basic Sciences, University of Global Health Equity, Kigali, Rwanda; ^14^ Institute of Biotechnology, Addis Ababa University, Addis Ababa, Ethiopia; ^15^ Department of Gynecology, Martin Luther University Halle-Wittenberg, Halle (Saale), Germany; ^16^ Institute of Medical Epidemiology, Biostatistics and Informatics, Martin Luther University Halle-Wittenberg, Halle (Saale), Germany

**Keywords:** breast cancer, estrogen receptor, immunohistochemistry, subtype, Ethiopia, Africa

## Abstract

**Purpose:**

Different biological characteristics, therapeutic responses, and disease-specific outcomes are associated with different molecular subtypes of breast cancer (BC). Although there have been different studies on BC in the Ethiopian capital city of Addis Ababa, there have been few studies in other parts of the nation, and none have evaluated biological characteristics in other locations in the context of the extensive ethnic and genetic diversity found in Ethiopia. This study was carried out to evaluate the distribution of immunohistochemistry (IHC) subtypes of BCs throughout four Ethiopian regions.

**Methods:**

A total of 227 formalin-fixed paraffin-embedded (FFPE) tissue blocks were collected from tertiary hospitals in four Ethiopian regions between 2015 and 2021. The IHC staining was performed for subtyping, ER, PR, HER2, and Ki-67 proliferation markers.

**Results:**

The mean age at diagnosis was 43.9 years. The percentage of ER and PR-negative tumors were 48.3% and 53.2%, respectively. The IHC subtypes showed the following distribution: 33.1% triple-negative breast cancer (TNBC), 27.6% luminal B, 25.2% luminal A, and 14.1% HER2 enriched. In multiple logistic regression analysis, grade III and HER2 positivity were associated with larger tumor size, and also originating from Jimma compared to Mekele.

**Conclusion:**

Patients with ER-negative, PR-negative, and TNBC were found in 48.3%, 53.2%, and 33.1% of cases, respectively, showing that half the patients could potentially benefit from endocrine treatment. A considerably high prevalence of TNBC was reported in our study, demanding additional research that includes genetic predisposition factors. Additionally, aggressive tumors were found in a high percentage of younger age groups, which must be considered when planning personalized treatment strategies.

## Introduction

In 2020, about 10 million people died due to cancer-related causes and the worldwide burden of cancer has significantly increased in recent years. In females, breast cancer (BC) is the most common malignant tumor, accounting for 11.7% of all cancer diagnoses, and is anticipated to cause more than 3 million new cases and 1 million fatalities by 2040 (1, 2). Of note, in Africa, 8.3% of newly diagnosed global BC cases are assumed and the amount of BC-related death is significantly higher (12.5%) when compared with other regions; 7.1% in North America and 6.4% in Western Europe (2). Additionally, the survival rate for BC patients in the continent is lower than the global average (3). Breast cancer has also been shown to occur at a younger age in African countries than in other regions, with a median age of about 45 years (4). Reports indicate that African women have a disproportionately high incidence of BC with poor prognosis, such as hormone receptor-negative, triple-negative, and basal phenotypic tumors (5).

In the past 20 years, molecular classification based on the expression of human epidermal growth factor receptor 2 (HER2), estrogen receptor (ER), progesterone receptor (PR), and Ki-67 has provided prognostic, predictive, and diagnostic information. The main subtypes that have been discovered are HER2-enriched (ER-, PR-, HER2+), luminal A (ER+, PR+, HER2-, and Ki-67< 20%), luminal B (ER+, PR+, HER2-, and Ki-67 ≥ 20% *or* ER+, PR+, and HER2+), and basal-like/triple-negative breast cancer (TNBC) (ER-, PR-, HER2-). These molecular subtypes are linked to distinct biological features, treatment responses, and disease-specific outcomes (6, 7), and show significant differences in the prediction of overall and disease-free survival (8).

In Ethiopia, BC is the most prevalent type of cancer, accounting for 31.9% of all female cancer cases, with 16,133 new cases and 9,061 fatalities (27.5%) in 2020 (1). In Ethiopia, the disease is diagnosed typically at an advanced stage and primarily affects young women in the country (9). Although different studies have been conducted on the incidence and molecular types of BC in the Ethiopian capital city of Addis Ababa, few studies have been done in multiple regions of the country and none has assessed several regions in the context of the extensive ethnic and genetic diversity found in Ethiopia (10, 11). Therefore, this study aimed to assess the distribution of immunohistochemistry-derived BC subtypes in several regions of Ethiopia.

## Methods

### Study area and samples

**Figure 1:**
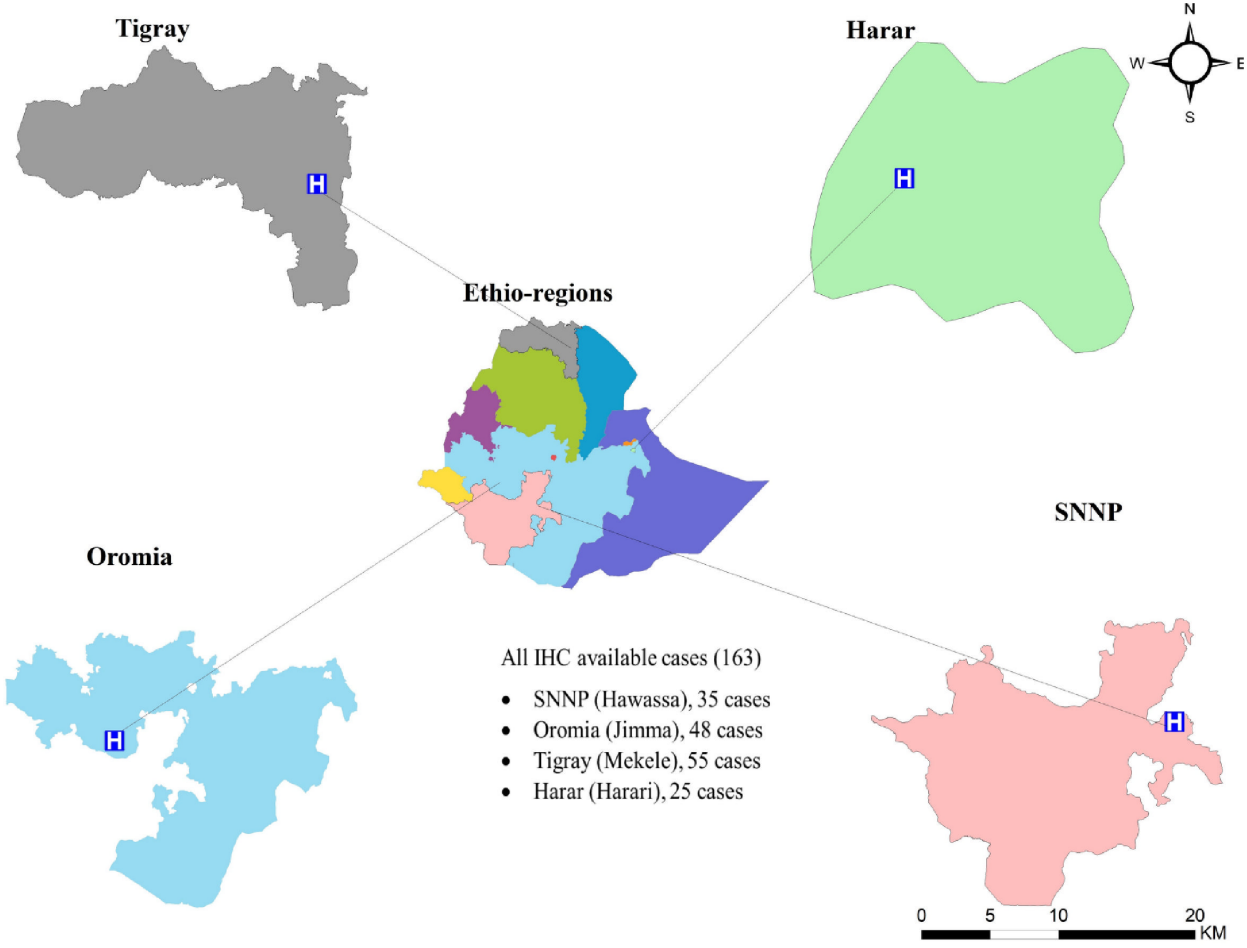
The study area and study centers (marked with an H to represent hospitals) location map.

A cross-sectional retrospective study involving 227 formalin-fixed paraffin-embedded (FFPE) tissue blocks were collected between 2015–2021 from four different regions: Hawassa Referral Hospital (Hawassa City, Southern Nations, Nationalities and Peoples (SNNP) region; n = 46), Jimma University Specialized Hospital (Jimma City, Oromia region; n = 53), Ayder Referral Hospital (Mekelle City, Tigray region; n = 95), and Hiwot Fana Specialized University Hospital (Harar City, Harar region; n = 33) (**Figure 1**). We selected these areas for our study because each has a main cancer treatment center, and may have a population with a diversified genetic makeup.

### Data collection

Demographic and histological data from the study hospitals were collected using a data collection form. The following data were collected: tumor size, histological grade, lymph node status, patient age, sex, and study sites. Information was derived from the pathology reports.

### Histopathological grade and stage

Histopathological grade and stage were determined using the Nottingham grading and TNM staging system, respectively (12, 13). Histopathological grade and type were checked and confirmed at the Armauer Hansen Research Institute and the Tikur Anbessa Specialized Hospital by three senior pathologists. Histopathology assessment on FFPE sections stained with hematoxylin and eosin was performed at AHRI to confirm the diagnosis.

### Immunohistochemistry

The IHC staining was performed on 227 FFPE tissue blocks using an optimized IHC protocol. Sections of FFPE tissue were cut at a thickness of 4 µm and rehydrated in water. Heat-induced epitope retrieval was carried out using Dako FLEX, a low pH retrieval buffer for Ki-67, and a high pH retrieval buffer for ER, PR, and HER2. The slides then underwent a 10-minute incubation with peroxidase-blocking solutions, followed by 30-minute incubations with specific primary antibodies and the EnVision FLEX/HRP. DAB chromogen was then applied for 5 minutes. The slides were counterstained for 30 seconds with hematoxylin and mounted with DPX and cover slip. Monoclonal mouse anti-human ER (DAKO clone Ep1; Agilent Technologies, Denmark) and anti-human PR (DAKO clone PgR636, Agilent Technologies, Denmark) antibodies were used for the staining. If a tumor exhibited 1% or more of tumor cell nuclear staining, it was considered to be ER/PR positive (14). The HER2/neu staining was performed using the HER2/neu reagent (Polyclonal, Agilent Technologies, Denmark). The grading expression was based on recommendations from Fitzgibbons et al. (2018): specimens scored as 0 or 1+ were classified as HER2/neu negative, and specimens scored as 3+ were considered positive. Specimens with a score of 2+ were considered equivocal (15). In multiple logistic regression analysis, the HER2/neu 2+ were excluded. According to the recommendation of the St. Gallen international panel of experts, a Ki-67 cut-off point of ≥ 20% was considered high (16). For ER and Ki-67 proliferation markers, we used DAKO mouse IgG1, Code X0931 negative control, and for PR and HER2, we used DAKO rabbit immunoglobulin fraction (solid-phase absorbed), Code X0936. Both were diluted to the same IgG concentration as the primary antibody. We used ductal epithelial cells from the breast as internal controls for ER and PR, and we used the mitotic index as an internal control for Ki-67 proliferation marker staining. Additionally, we performed positive controls using normal endometrial stroma for PR, cervix epithelial cells for ER, and tonsil for Ki-67 in each experiment. We optimized our HER2 positive control using our BC samples. IHC tests were conducted in a centralized AHRI laboratory using archival blocks collected from each Hospital.

### IHC subtyping

Breast cancer subtyping in this study was performed based on the consensus of St. Gallen international experts that divided BC into the following four subtypes: luminal A (ER and/or PR-positive, HER2-negative, and Ki-67 < 20%), luminal B (ER and/or PR-positive, HER2-positive or ER- and/or PR-positive, HER2-negative, and Ki67 ≥ 20%), HER2-enriched (ER- and PR-negative, HER2-positive), and triple-negative (ER-, PR-, and HER2-negative) (16).

### Data analysis

Data collected from the pathology report and IHC results were entered and analyzed using SPSS Version-25 software. Univariate Chi-square tests were used to assess the hypothesis of the association between predictor and outcome variables of interest. Logistic regression was performed to determine associations between a given predictor and outcome variables after correcting for the effects of all other predictors. Statistical significance was defined as a p-value less than 0.05.

## Results

### Demographic and histopathological characteristics

In this study, 227 tumor specimens were collected. The mean age at diagnosis was 43.9 (SD = 13.9) years. The average age of Hawassa study site’s breast cancer patient was lower than those of other research sites (38.7 years) (**Table 1**).

**Table 1:** Basic demographic information of the study population at four study sites.

Variables		Frequency (%)	Study sites			
			Hawassa	Jimma	Mekele	Harar
**Age**	**< 50 years**	151 (66.5)	40 (87.0)	31 (58.5)	59 (62.1)	21 (63.6)
	**≥ 50 years**	76 (33.5)	6 (13.0)	22 (41.5)	36 (37.9)	12 (36.4)
	**Total**	227 (100.0)	46 (100.0)	53 (100.0)	95 (100.0)	33 (100.0)
	**Mean age** ± SD	43.9 ± 13.9 years	38.7± 11.7 years	44.9± 13.9 years	45.2± 14.2 years	44.7± 13.6 years
**Sex**	**Female**	216 (95.2)	45 (97.8)	51 (96.2)	89 (93.7)	30 (93.9)
	**Male**	11 (4.8)	1 (2.2)	2 (3.8)	6 (6.3)	11 (4.8)
	**Total**	227 (100.0)	46 (100.0)	53 (100.0)	95 (100.0)	33 (100.0)

Tumor size greater than 5 cm (T3) at the time of diagnosis accounted for 28.9% of the cases, with a higher percentage (48.9%) in southwest Ethiopia (Jimma). Any tumor size growing into the chest or skin (T4) was high in Harer (42.3%). Involvement of the lymph node was found in 63.7% of cases, with a higher percentage in northern Ethiopia (Mekele) (75.8%). Histological grades II and III accounted for 66% of the cases. Age, tumor size, and histologic grade were all substantially associated with study sites, with younger cases in southern Ethiopia (Hawassa), larger tumor size in southwestern Ethiopia (Jimma), and higher histological grade in northern Ethiopia (Mekele) (**Table 2**). Invasive ductal carcinoma was the most common histomorphologic type of BC (84.0%), followed by invasive lobular carcinoma.

**Table 2:** Distribution of histopathological, and immunohistochemical characteristics of breast cancer at the four study sites.

Variables*		Frequency (%)	Study sites				p-value
			Hawassa	Jimma	Mekele	Harar	
**Tumor Size**	**T1**	16 (8.0)	2 (4.9)	2 (4.3)	10 (11.5)	2 (7.7)	0.001
	**T2**	77 (38.3)	14 (34.1)	9 (19.1)	43 (49.4)	11 (42.3)	
	**T3**	58 (28.9)	13 (31.7)	23 (48.9)	20 (23.0)	2 (7.7)	
	**T4**	50 (24.9)	12 (29.3)	13 (27.7)	14 (16.1)	11 (42.3)	
	**Total**	201 (100.0)	41 (100.0)	47 (100.0)	87 (100.0)	26 (100.0)	
**Grade**	**I**	74 (33.9)	18 (39.1)	25 (47.2)	25 (26.3)	9 (27.3)	0.033
	**II**	70 (32.1)	16 (34.8)	16 (30.2)	25 (26.3)	12 (36.4)	
	**III**	74 (33.9)	12 (26.1)	12 (22.6)	45 (47.4)	12 (36.4)	
	**Total**	218 (95.6)	46 (100.0)	53 (100.0)	95 (100.0)	33 (100.0)	
**Lymph node status**	**Positive**	123 (63.7)	26 (56.5)	30 (56.6)	50 (75.8)	16 (59.3)	0.088
	**Negative**	70 (36.3)	20 (43.5)	23 (43.4)	16 (24.2)	11 (40.7)	
	**Total**	192 (100.0)	46 (100.0)	53 (100.0)	66 (100.0)	27 (100.0)	
**ER**	**Positive**	104 (51.7)	22 (48.9)	27 (51.9)	40 (52.6)	15 (53.6)	0.976
	**Negative**	97 (48.3)	23 (51.1)	25 (48.1)	36 (47.4)	13 (46.4)	
	**Total**	201 (100.0)	45 (100.0)	52 (100.0)	76 (100.0)	28 (100.0)	
**PR**	**Positive**	94 (46.8)	23 (51.1)	20 (38.5)	41 (53.9)	10 (35.7)	0.193
	**Negative**	107 (53.2)	22 (48.9)	32 (61.5)	35 (46.1)	18 (64.3)	
	**Total**	201 (100.0)	45 (100.0)	52 (100.0)	76 (100.0)	28 (100.0)	
**HER2**	**Positive**	42 (22.0)	8 (18.2)	11 (21.6)	13 (19.1)	10 (35.7)	0.269
	**Negative**	128 (67.0)	30 (68.2)	38 (74.5)	45 (66.2)	15 (53.6)	
	**Equivocal**	21 (11.0)	6 (13.6)	2 (3.9)	10 (14.7)	3 (10.7)	
	**Total**	191 (100.0)	44 (100.0)	51 (100.0)	68 (100.0)	28 (100.0)	
**Ki-67**	**Ki-67 < 20%**	106 (57.0)	22 (56.4)	40 (81.6)	37 (52.9)	7 (25.0)	< 0.0001
	**Ki-67 ≥ 20%**	80 (43.0)	17 (43.6)	9 (18.4)	33 (47.1)	21 (75.0)	
	**Total**	186 (100.0)	39 (100.0)	49 (100.0)	70 (100.0)	28 (100.0)	
**Subtype**	**Luminal A**	41 (25.2)	12 (34.3)	17 (35.4)	9 (16.4)	3 (12.0)	0.114
	**Luminal B**	45 (27.6)	8 (22.9)	9 (18.8)	19 (18.8)	9 (36.0)	
	**HER2 enriched**	23 (14.1)	6 (17.1)	4 (8.3)	7 (12.7)	6 (24.0)	
	**TNBC**	54 (33.1)	9 (25.7)	18 (37.5)	20 (36.4)	7 (28.0)	
	**Total**	163 (100.0)	35 (100.0)	48 (100.0)	55 (100.0)	25 (100.0)	

*Variables are only shown for cases with known results. Differences of features among study sites assessed by X^2^ test.

### Tumor size

In univariate analysis, tumor size was determined in 201 cases (26 cases were missed), and Jimma was the region with the highest percentage of T3 and T4 tumors (76.6%) (**Table 2**). For multiple logistic regression analysis, 157 cases were included. For these analyses a binary outcome variable for tumor size was created by summing T3 and T4 tumors within a large category and T1 and T2 tumors for a small category. Grade III tumors were 2.5 times more likely than grade I or II tumors to have a large (T3 or T4) tumor size (*p* = 0.025). The HER2-positive tumors were 4.1 times more likely than HER2-negative tumors to have a large (T3 or T4) tumor size (*p* = 0.007). Breast cancer cases from the south (Hawassa) and southwest (Jimma) were 3.1 and 7.7 times, respectively, more likely to have T3 or T4 tumors than those from the north (Mekele) (**Table 3**).

**Table 3:** Multiple logistic regression analysis of demographic and histopathological parameters predicting tumor size.

Parameters*		All N (%) = 157	Large tumor size (T3 and T4) (n = 95) vs small tumor size (T1 and T2) (n = 62).	
			OR (95% CI)	p-value
**Age group (years)**	**< 50 (ref) #**	107 (68.2%)	1.40 (0.61–3.19)	0.430
	**≥ 50**	50 (31.8%)		
**Grade**	**I or II (ref)**	102 (65.0%)	2.57 (1.13–5.84)	0.025
	**III**	55 (35.0%)		
**Lymph node involvement**	**Yes**	102 (65.0%)	1.60 (0.75–3.45)	0.228
	**No (ref)**	46 (35.0%)		
**HER2**	**Negative (ref)**	103 (65.6%)		
	**Positive**	34 (21.7%)	4.14 (1.47–11.67)	0.007
	**Equivocal**	20 (12.7%)	2.02 (0.70–5.81)	0.193
**Study sites**	**Hawassa**	40 (25.5%)	3.10 (1.13–8.47)	0.028
	**Jimma**	45 (28.7%)	7.7 (2.65–22.77)	< 0.0001
	**Harar**	22 (14.0%)	1.47 (048–4.57)	0.502
	**Mekele (ref)**	50 (31.8%)		

*Tumor size was the outcome variable. Binary categories of large (T3 or T4) and small (T1 or T2) were created with large tumor size as the reference value. The indicated dependent variables or parameters are listed in the left column. # The reference values for the predictor variables are indicated within parentheses.

### Estrogen receptor, HER2, and Ki-67 proliferation

In univariate analysis, 201 cases were analyzed (26 cases were missed), and half of the specimens were ER- and PR-negative (**Table 2**). In this study, 161 BC cases were included in multiple logistic regression analysis, and the presence of an ER-positive tumor with a histological grade I or II was 2.9 times more common than that of a grade III tumor (*p* = 0.005). The chance of having ER-positive breast cancer appears to be 2.1 higher in older women (>50 years vs. <50 years) (*p* = 0.039) (**Table 4**).

**Table 4:** Multiple logistic regression analysis of positive ER and HER2 status, and Ki-67 ≥ 20% with other variables among 161 (ER), 137 (HER2), and 149 (Ki-67) study participants.

Parameters*		ER-positive (n = 79) ER-negative (n = 82)		HER2-positive (n = 34) HER2-negative (n = 103)		Ki-67 ≥ 20% (n = 59) Ki-67 < 20% (n = 90)	
		OR (95% CI)	p-value	OR (95% CI)	p-value	OR (95% CI)	p-value
**Age group (years)**	**< 50 (ref)#**	2.18 (1.04–4.58)	0.039	0.61 (0.2–1.61)	0.317	1.65 (0.70–3.90)	0.253
	**≥ 50**						
**Grade**	**I or II**	2.96 (1.40–6.26)	0.005	1.18 (0.47–2.96)	0.727	0.20 (0.08–0.46)	<0.0001
	**III (ref)**						
**Tumor Size**	**T1 or T2 (ref)**	0.98 (0.49–1.98)	0.956	3.85 (1.39–10.68)	0.010	0.82 (0.79–4.15)	0.158
	**T3 or T4**						
**Lymph node involvement**	**Yes**	1.60 (0.80–3.20)	0.185	0.99 (0.41–2.41)	0.981	0.67 (0.230–1.51)	0.337
	**No (ref)**						
**Study sites**	**Hawassa**	1.18 (0.48-2.93)	0.719	0.82 (0.23-2.92)	0.760	1.69 (0.58-4.91)	0.333
	**Jimma**	0.71 (0.29-1.76)	0.461	1.04 (0.32-3.43)	0.947	0.42 (0.14-1.25)	0.118
	**Harar**	1.51 (0.51–4.47)	0.456	3.61 (1.01–12.87)	0.048	6.39 (1.85–22.09)	0.003
	**Mekele (ref)**						

*Binary logistic regression was performed with ER, HER2 positivity and Ki-67 ≥ 20% marker as the outcome variables (with marker negativity and Ki-67 < 20% as the reference value), and predictor variables listed in the parameter column at left.

The reference values for the predictor variables are indicated within parentheses.

Among a total of 191 specimens (36 cases were missed) included in univariate analysis, 22% of the cases were HER2 positive, with the highest percentage (35.7%) reporting from eastern Ethiopia (Harar) (**Table 2**). In the study of 137 BC cases that were included for multiple logistic regression analysis, T3 or T4 tumors were 3.8 times higher than T1 or T2 tumors to be HER2-positive (*p* = 0.01). Additionally, BC cases in eastern Ethiopia (Harer) were 3.6 times more likely than cases in northern Ethiopia (Mekele) to be HER2 positive (**Table 4**).

In univariate analysis, a total of 186 cases of BC were analyzed (41 cases were missed), and the Ki-67 scores of ≥ 20% were observed in 43.0% of BC cases (**Table 2**). In the multiple logistic regression analysis of 149 BC patients, eastern Ethiopia (Harer) was 6.4 times more likely than northern Ethiopia (Mekele) to have Ki-67 ≥ 20% (**Table 4**).

### IHC subtypes distribution

In univariate analysis, 163 samples with all IHC available the IHC subtypes showed the following distribution: 33.1% TNBC, 27.6% luminal B, 25.2% luminal A, and 14.1% HER2 enriched (**Table 2**). Among the 131 BC patients included in multiple logistic regression analysis, luminal A subtypes were 10.4 times more likely to have histological grade I or II than grade III (*p* = 0.002). The luminal A subtype of BC in southern Ethiopia (Hawassa) was 3 times more likely than in northern Ethiopia (Mekelle) (*p* = 0.109). We observed cases with tumor size T3 or T4 were 4.8 times higher to have HER2 enriched subtypes than tumor size T1 or T2 (**Table 5**). In univariate analysis, TNBC was found in the highest number of cases from southwestern Ethiopia (Jimma) (37.5%), followed by cases from northern Ethiopia (Mekele) (36.4%) (**Table 2**). Using a multiple logistic regression model, after controlling for other variables, TNBC in southwestern Ethiopia (Jimma) was 2.1 times more likely than in northern Ethiopia (Mekelle), though this did not reach statistical significance (*p* = 0.18) (**Table 5**).

**Table 5:** Multiple logistic regression analysis of demographic and histopathological parameters, taken as predictive variables for individual IHC subtypes compared to others (N = 131).

Parameters		All N (%) = 131	Luminal A (n = 30)		Luminal B (n = 37)		HER2-enriched (n = 19)		TNBC (n = 45)	
			OR (95% CI)	p-value	OR (95% CI)	p-value	OR (95% CI)	p-value	OR (95% CI)	p-value
Age group (years)	< 50	90 (68.7%)	0.42 (0.15–1.19)	0.105	0.95 (0.39–2.328)	0.906	1.48 (0.44–5.03)	0.527	1.48 (0.62–3.57)	0.382
	≥ 50 (ref)	41 (31.3%)								
Tumor size	T1 or T2 (ref)	51 (38.9%)	0.52 (0.20–1.38)	0.188	1.16 (0.49–2.73)	0.742	4.84 (1.23–19.03)	0.024	0.61 (0.25–1.47)	0.267
	T3 or T4	80 (61.1%)								
Grade	I or II	85 (64.9%)	10.43 (2.36–55.39)	0.002	1.64 (0.67–4.03)	0.280	0.72 (0.24–2.14)	0.554	0.22 (0.09–0.54)	0.001
	III (ref)	46 (35.1%)								
Lymph nodes involved	Yes	86 (65.6%)	1.04 (0.39–2.72)	0.944	1.34 (0.57–3.14)	0.507	1.33 (0.42–4.18)	0.628	0.66 (0.29–1.65)	0.336
	No (ref)	45 (34.4%)								
Study sites	Hawassa	31 (23.7%)	2.94 (0.79–10.93)	0.109	0.76 (0.25–2.38)	0642	1.03 (0.24–4.46)	0.968	0.53 (0.17–1.71)	0.290
	Jimma	42 (32.1%)	1.829 (0.35–4.80)	0.702	0.55 (0.17–1.72)	0.302	0.49 (0.11–2.28)	0.363	2.13 (0.71–6.41)	0.180
	Harar	20 (15.3%)	0.37 (0.06–2.27)	0.284	1.87 (0.59–5.99)	0.290	2.18 (0.49–9.67)	0.304	0.41 (0.10–1.63)	0.205
	Mekele (ref)	38 (29.0%)								

## Discussion

Immunohistochemical markers are frequently used to guide treatment choices, classify BC into biologically distinct subtypes, and serve as prognostic and predictive markers (17). The IHC staining procedures to determine therapeutic biomarkers status have recently been introduced into clinical practice in Ethiopia but are still not available in all regions of the country. We chose these study sites because genetic research, despite its lack of specificity, has demonstrated that Ethiopian genetic diversity reflects linguistic stratification and diverse influences on the Ethiopian gene pool (11). Our research was conducted in regional areas of Ethiopia with oncology care only recently initiated. This study found a high proportion of BCs with advanced clinical and pathologic characteristics, such as a high prevalence of lymph node involvement, large tumor size, and high histological grade. The percentage of ER- and PR-negative results reported in this study was higher than in earlier Ethiopian studies (18–22). The TNBC was seen to be more frequent in southwest Ethiopia (Jimma) and north Ethiopia (Mekele). Study sites showed a different composition of age groups, tumor size, histological grade, and Ki-67 proliferation index.

In this study, the mean age for BC patients at diagnosis was 43.9 years. Most patients were premenopausal (younger than 50 years old), with the highest frequency (87.0%) in Hawassa. A relatively young age at presentation is comparable to other earlier studies carried out in Ethiopia, which reported patients with mean ages of 43 to 47 years (19–21) and other African studies reported mean ages less than 50 years of age (23–31). On the other hand, in Europe, the mean age is significantly higher; 62.7 years in Switzerland (32) and 63.5 in Sweden (33). This distinction was also revealed by a comparative study, where patients from Sudan were 10 years younger than those from Germany and Italy (34, 35), and patients in Nigeria were 21 years younger than those in the UK (36). This is possibly due to the young population structure in Ethiopia and Africa, with a predominance of people below the age of 60 years.

Histological grades II and III were found in the majority of patients in the current study, with the highest proportion in northern Ethiopia (Mekele). A considerable percentage of cases (53.8%) had tumors that were T3 or T4, with southwestern Ethiopia (Jimma) reporting the largest number of cases (75.6%). In this study, lymph nodes were involved in 63.7% of BC patients, with northern Ethiopia (Mekele) having the highest frequency (75.8%). The histological grade is now taken into account when selecting the therapy strategy (37). This study agrees with other studies reported in Ethiopia and other African countries such as Kenya, Ghana, the Republic of Congo, Ivory Coast, Egypt, Libya, and Malawi (19, 20, 24, 27, 31, 38–42). Compared with European cohorts, grade I tumors were most common in Switzerland (32) and Belgium (43). Lack of knowledge and awareness of early detection, poor perception of BC, lack of financial and social support, absence of adequate population screening, poor support system, and sociocultural factors including tradition, belief, and fear all contribute to the severity of BC in Africa (44, 45). According to a study done in Ethiopia, women hide tumors from their families because a mastectomy is related to a perception of premature death, infertility, and divorce (45). In the present study, a high proportion of patients under the age of 50 years, a high degree of lymph node involvement, and a high degree of Ki-67 related proliferation all suggest that appropriate chemotherapy should be initiated in these settings with limited resources. These tumor features may increase cancer mortality, demanding a comprehensive approach that includes raising cancer awareness, upgrading cancer infrastructure, and providing prompt treatment.

Breast cancer histomorphological characteristics have been well-documented as a significant prognostic factor. By far, the most common is invasive carcinoma of no special type (NST). The other forms of BC have slightly better outcomes (46–48). The most prevalent histomorphologic type of BC in the current study is NST, accounting for 84% of the cases. A similar finding is reported in Ethiopia and other countries (19, 25, 27, 38, 41, 49–52).

Molecular subgroups were also significant predictors of BC mortality (53). Poorer outcomes have been linked to the triple-negative and HER2 subtypes (54). TNBC has a poor prognosis, high levels of invasiveness, and metastatic potential. Additionally, they are resistant to endocrine- and HER2-targeted therapies (55). A higher percentage of TNBC subtypes (33.1%) was reported in this study, with the highest percentage in southwest Ethiopia (Jimma) (37.5%), followed by north Ethiopia (Mekelle) (36.4%), which is higher than the 23% (21, 56) and 24.8% (18) reported in earlier Ethiopian studies from the capital city. TNBC subtypes were found on average in 26.4% of patients from African countries, with 22.8% in East Africa, 14.9% in Middle Africa, 22.6% in North Africa, and 16.6% in South Africa. However, west Africa had a substantially higher rate, accounting for 45.7% (57). The percentage of TNBC is substantially lower in Europe, the UK (0.3%) (58), and Italy (8.1%) (59). Comparative research showed Sudan had a TNBC rate of 34.5%, while Germany had a rate of 14.2% (34). Compared to populations of European heritage, populations of African descent had the greatest reported prevalence of TNBC (60). One important factor is the higher prevalence of TNBCs in younger age groups. Additionally, this could be explained by hereditary factors, such as the founder BRCA gene mutation (61, 62), not reported yet from Ethiopia. Another study also reveals the connection between African ancestry and the immunologic profile of TNBC (63).

Luminal A subtypes have the best prognosis, and the most effective therapy for this subtype is tamoxifen or aromatase inhibitors (64). Luminal B subtypes are more severe and have a worse prognosis than Luminal A subtypes (64). In the present study, the percentage of luminal B BC was 27.6%, which is comparable with a prior study in Ethiopia, where it was 26% (21). In this study, the percentage of luminal A subtype was 25.2%. An earlier study conducted in Ethiopia found a higher proportion of luminal A at 40% (21). The percentage of luminal A breast subtype is much higher in Europe; 73.2% in Switzerland (32) and 70.3% in Italy (59). The comparative study conducted between Africa and Europe also showed a higher luminal A subtype in Leuven than in Kinshasa with 64.5% and 40.2%, respectively (43). Another study also reported a higher percentage of luminal A in Germany than in Sudan with 68.4% and 36.9%, respectively (34). This is probably due to the lack of the older age group who have a high proportion of luminal A subtypes in the African setting.

The HER2-enriched BC subtype is more aggressive and has a worse prognosis than luminal subtypes (64), especially before the availability of modern HER2 neu-directed therapies. The development of anti-HER2-targeted drugs has significantly increased patient survival rates for this subtype (65). The current study found 14.1% of BCs to be HER2-enriched subtypes, which is greater than the 10% (21) and 9.5% (18) found in an earlier study in Ethiopia. In comparison to the present, lower percentages of the HER2-enriched subtype of BC was observed in the UK (9.1%) (58), Italy (6.0%) (59), and Switzerland (5.6%) (32). A comparative study revealed that the HER2-enriched subtype is higher in Sudan (15.7%) than in Germany (6.8%) (34). We had 11% of cases that were HER2+ or equivocal, a substantial proportion. We did not perform fluorescent *in-situ* hybridization (FISH) for equivocal cases; however, we recommend that FISH should be performed in a future investigation to determine the precise number of HER2-enriched BC subtypes. This study provides important data that can be used to advocate for the appropriate allocation of resources to support developing pathology capacity. This is particularly timely, as the patents of the technology backbone for HER2-directed therapies have expired and global access to HER2 neu-directed therapies are expected to increase.

Endocrine therapy is a significant part of treatment for BCs that are ER-positive (66). Tamoxifen and an aromatase inhibitor should be a regular component of endocrine therapy for the majority of postmenopausal and premenopausal women with receptor-positive BC, respectively (67). The 15-year mortality rates of BC were reduced by around 30% and 40% by tamoxifen and aromatase inhibitors in adjuvant settings, respectively (68). ER-positivity is detected in 51.7% of the patients in the current study. A higher percentage of ER-positivity, with values of 65.5% (19), 73% (20), 65% (21), and 65.3% (22), were observed in prior Ethiopian studies. In a systematic review from sub-Saharan Africa, 42% of BC cases were ER-positive, with 35.0% in West Africa (69). Higher rates of ER-positive BC were reported in other nations than the present study: 75.7% in Saudi Arabia (70), 87.9% in Sweden (33), 85.3% in Switzerland (32), 84% in Norway (71), 99.3% in the UK (71), and 76% in the USA (72). There was a significant correlation between histological grade and ER status, with a higher histological grade more likely to be ER-negative, in this study and confirmed by other studies (73). Based on our findings, receptor testing availability should be a priority to offer the best treatment for BC patients.

## Conclusion

A high proportion of BCs with advanced clinical and pathologic characteristics, such as extensive lymph node involvement, large tumor size, and high histological grade were found in this study, pointing to the certain need for chemotherapy for the majority of patients. Half the patients were ER-positive in this study, indicating that receptor status testing and availability of endocrine treatment need to be prioritized in cancer control programs.

TNBC was reported with higher frequency in southwest Ethiopia (Jimma) and northern Ethiopia (Mekele) compared to the other regions. A different pattern of age, tumor size, histological grade, and Ki-67 proliferation index was found between the study sites, showing the need for each tertiary center to monitor the composition of features among their respective patients. The considerably high rates of TNBC and hormone receptor-negative tumors (still showing half the patients with endocrine-sensitive disease) in our study need special attention. Such a variety of features need close collaboration between surgeons, oncologists, pathologists, radiologists, and radiotherapists, in addition to linkage to lower-level health facilities. Individual treatment recommendations should be discussed in interdisciplinary tumor boards and offered to the patients. Especially utilization of adequate imaging, neoadjuvant chemotherapy, and specialized surgery is needed. In this study we have focused primarily on endocrine markers because of the implication in therapy. A number of other biomarkers (including Bcl-2, GCDFP-15, TRPS1, Cytokeratins and others) have been studied, showing diagnostic or prognostic promise. Future studies with these and other markers as well as genetic mutational analysis are planned”.

## Limitations

The small sample size, retrospective nature, and absence of analysis of HER2 equivocal data using fluorescent *in-situ* hybridization is the major limitation of this work. Larger studies in the future studies to solidify our study findings are warranted. We were not able to perform FISH on the HER2 equivocal cases (11% of total) for which this procedure is indicated. There was a lack of ability to ensure that the pre-analytical variables were optimized or standardized across these sites. We see strength in performing centralized IHC for all samples of regions that have not been studied before.

## Data availability statement

The raw data supporting the conclusions of this article will be made available by the authors, without undue reservation.

## Ethics statement

Ethical approval was obtained from the College of Natural Science Institutional Ethics Review Board (CNS-IRB) Addis Ababa University (No. IRB/032/2018) and AHRI/ALERT Ethics Review Committee (AAERC) (No. PO/27/19). Patients' informed consent was not required because we used archived tissue blocks.

## Author contributions

EB contributed to study design, sample and data acquisition, analysis, interpretation, and writing of the original and final draft. DD, TG, SA, MY, BF, AlA, AdA, AbA, TD, and SG contributed to data analysis, data interpretation, sample acquisition and experimental work. MC, DB, LT, MB, and DS contributed to data analysis, data interpretation, experimental work and editing of the manuscript. AD, DM, TS, EK, and RH contributed to the study design, data acquisition, data analysis, data interpretation, and editing of the manuscript. All authors contributed to the article and approved the submitted version.
